# Prevalence of Sleep Disorders Among Patients With Type 2 Diabetes Mellitus at Primary Healthcare Centers in the South Region of Abha City

**DOI:** 10.7759/cureus.44749

**Published:** 2023-09-05

**Authors:** Ali Al Amri, Mohammed Abdullah A Alshahrani, Mousa A Asiri, Majdoleen A Abdulrahman, Ali Yahya A Alshehri, Meshal Mohammed M Alqahtani, Abdulkhaliq Abdullah A Oraydan, Sarah I Summan, Thekra S Alqahtani, Aljohrah M Al Hunaif

**Affiliations:** 1 Family Medicine, Asir Central Hospital, Abha, SAU; 2 Medicine and Surgery, King Khalid University, Abha, SAU; 3 Technician Pharmacy, Primary Care Center Al Mansak, Abha, SAU

**Keywords:** risk factors, sleep quality, prevalence, sleep-disorder, type 2 diabetes

## Abstract

Background

Type 2 diabetes is a chronic condition that affects the way the body processes blood sugar (glucose). This issue is of considerable importance in the field of public health, as it has a global impact on a substantial number of individuals. The primary emphasis in the management of type 2 diabetes is centered around achieving glycemic control, implementing lifestyle adjustments, and employing pharmaceutical therapies as preventive measures or for the purpose of managing problems that may arise as a result of the disease.

Aim

This research aimed to investigate the prevalence of sleep-belated issues among individuals diagnosed with type 2 diabetes.

Methodology

A total of 230 participants with type 2 diabetes patients of primary healthcare in Abha city whose age is ≥18 years were included in the study. The data collection process involved the distribution of a self-administered questionnaire that assessed various aspects of sleep disturbances, including difficulties in falling asleep, waking up during the night, excessive daytime sleepiness, and restless legs or leg muscle cramps. The questionnaire also collected demographic information and data on potential risk factors such as alcohol consumption, caffeine consumption, and smoking/tobacco product use. Data analysis was conducted using chi-square tests and significance levels were set at p < 0.05.

Results

The findings revealed a prevalence of sleep disturbances among individuals with type 2 diabetes. Difficulties in falling asleep and waking up during the night were reported by a substantial proportion of participants, and a notable number experienced excessive daytime sleepiness. Restless legs or leg muscle cramps that interrupted sleep were experienced occasionally by 16.5% and frequently by 8.7% of the participants. The study also found a significant association between the presence of sleep problems and lower sleep quality ratings. However, no significant associations were found between sleep disturbances and the duration of type 2 diabetes or the examined risk factors.

Conclusion

The findings from this study emphasize the detrimental effects of sleep disturbances on sleep quality and suggest that improving sleep quality can positively influence the overall health and well-being of individuals with type 2 diabetes.

## Introduction

Type 2 diabetes (T2D) is a chronic condition that affects the way the body processes blood sugar (glucose) [1]. Prevalence is a measure of the frequency of a disease or health condition in a population at a particular point in time. This issue is of considerable importance in the field of public health, as it has a global impact on a substantial number of individuals. The primary emphasis in the therapy of T2D is on achieving glycemic control, implementing lifestyle adjustments, and utilizing pharmaceutical interventions to prevent or effectively address the complications that are commonly associated with this condition [2]. Nevertheless, recent research indicates that patients diagnosed with T2D frequently encounter disruptions in their sleep patterns, which can potentially have adverse consequences on their general health and quality of life [3]. Sleep disturbances involve a variety of sleep-related issues, such as challenges in beginning or sustaining sleep, sleep that does not provide adequate restoration, excessive tiredness during the day, sleep apnea, and restless legs syndrome [4]. Sleep disorders can result in fragmented sleep, diminished sleep quality, and insufficient sleep length, impacting all facets of persons' life. Sleep disorders have wide-ranging effects that go beyond personal perceptions of sleep quality. Research has shown that these disturbances are linked to negative health consequences, such as impaired management of blood sugar levels, heightened risk of cardiovascular disease, limited cognitive abilities, and reduced overall well-being [5].

Gaining a comprehensive understanding of the frequency and consequences of sleep disorders in patients diagnosed with T2D is of paramount importance in order to enhance their overall health outcomes and improve their quality of life. Through an examination of the correlation between sleep disruptions and the occurrence of T2D, healthcare practitioners and researchers have the opportunity to devise focused interventions aimed at mitigating these concerns and enhancing the overall management of diabetes. The primary objective of this study is to examine many crucial factors pertaining to sleep disorders among persons diagnosed with T2D. This study aims to ascertain the prevalence of sleep disorders, facilitating a full comprehension of the frequency of these concerns among the targeted group. Furthermore, this study aims to investigate the potential bidirectional correlation between sleep disorders and glycemic control, with a specific focus on examining the influence of inadequate sleep on blood glucose regulation. In addition, this study will evaluate the effects of sleep disorders on the overall quality of life and well-being of patients with T2D, taking into account the various physical, emotional, and social ramifications that may arise. Finally, this study aims to identify potential risk factors linked to sleep disturbances in the target group, including obesity, duration of diabetes, and the presence of comorbidities. The identification of these risk factors will enable the identification of individuals at high risk and inform the development of intervention options. This study aims to make a scholarly contribution to the existing literature on sleep disorders among patients diagnosed with T2D by addressing the specified research objectives.

Sleep disorders are commonly observed in patients diagnosed with T2D, and the implications of these disruptions can have wide-ranging effects. Numerous research investigations have been conducted to examine the frequency of sleep disruptions among persons diagnosed with T2D, uncovering concerning levels. An investigation conducted by Hashimoto et al. [6] revealed that around 66.4% of persons diagnosed with T2D reported encountering various sleep disturbances, including challenges in initiating or sustaining sleep, non-restorative sleep, and excessive daytime sleepiness. The observed prevalence of this phenomenon is notably greater in comparison to the broader population, underscoring the necessity for additional inquiry. One of the primary indicators of diabetes treatment is poor glycemic control, which is characterized by increased levels of blood glucose [7]. Numerous investigations have provided evidence of a reciprocal association between sleep disorders and glycemic regulation. According to a study, individuals diagnosed with T2D who encountered disruptions in their sleep patterns demonstrated inferior glucose control in comparison to those individuals who did not experience any sleep disturbances [7]. On the other hand, studies have demonstrated that inadequate management of glucose levels can contribute to disturbances in sleep patterns, establishing a detrimental cycle.

In addition, sleep disorders exert a substantial influence on the quality of life and general welfare of patients diagnosed with T2D. There is a correlation between inadequate sleep duration and poor sleep quality, and several negative consequences have been observed as a result. These consequences include a decline in quality of life, heightened levels of daytime weariness, diminished cognitive abilities, and poorer psychosocial functioning [8]. In a cross-sectional study conducted by Lee et al. [9], it was shown that persons diagnosed with T2D and experiencing sleep difficulties had worse scores on health-related quality-of-life assessments in comparison to individuals without sleep disorders. The aforementioned results emphasize the significance of incorporating interventions for sleep disruptions as an essential component of diabetes care.

It is imperative to identify potential risk factors that are linked to sleep disturbances in patients diagnosed with T2D in order to develop interventions that are specifically tailored to address these concerns. Numerous factors have been implicated in the etiology of sleep disruptions. The presence of obesity, a frequently occurring comorbidity in individuals with T2D, has consistently demonstrated an association with sleep problems, particularly obstructive sleep apnea (OSA) [10]. Furthermore, previous studies have indicated that the length of time an individual has been diagnosed with diabetes, as well as the coexistence of other medical conditions like cardiovascular disease or depression, may contribute to an increased likelihood of experiencing sleep disturbances [11]. The comprehension of these risk variables can facilitate the formulation of customized approaches for enhancing sleep health in patients diagnosed with T2D.

The results of this study will provide valuable insights for healthcare practitioners, researchers, and policymakers regarding the significance of including sleep health in the overall management of diabetes. In conclusion, the enhancement of sleep health in patients diagnosed with T2D holds the capacity to optimize glycemic control, mitigate the likelihood of complications, and increase general well-being, hence resulting in improved health outcomes for this specific demographic.

## Materials and methods

Survey design

A cross-sectional survey design was employed to investigate the prevalence of sleep-related issues among individuals with T2D whose age is ≥18 years to explore their relationship with glycemic control, lifestyle factors, and potential risk factors. The survey was designed as a self-administered questionnaire to gather information from participants about various factors related to sleep quality and diabetes management. Non-type 2 diabetic patients, type 2 diabetics with local or systemic illness that would confound the result of the study, non-residents of Abha city, diabetic patients with psychological disorders, and pregnant women were all excluded from this study.

Procedure

A total sample size of 230 participants was captured in the survey. The data collection process involved the distribution of the self-administered questionnaire through a social media platform (WhatsApp) to ensure easy computation of the respondents’ data. Participants were recruited through online diabetes support groups, forums, and diabetes-focused social media pages. Ethical considerations were considered, ensuring informed consent from participants and protecting their privacy and confidentiality.

Instruments

The primary instrument used in this study was the Pittsburgh Sleep Quality Index (PSQI) which was modified to fit the local language of the population under study. The PSQI is a widely used and validated self-report questionnaire designed to assess sleep quality and disturbances over a one-month time interval. In addition to the PSQI, the questionnaire included items related to demographic information and variables relevant to sleep quality, such as age, gender, duration of T2D, use of diabetes medications, the presence of other medical conditions affecting sleep quality, lifestyle factors (e.g., physical activity, caffeine consumption, tobacco use), and the use of electronic devices before bedtime. These variables were included to identify potential risk factors associated with sleep disturbances in individuals with T2D.

Data analysis

Python Jupyter Notebook version 6.4.5 was utilized for the purpose of data analysis. The chi-square (χ^2^) test was employed to analyze categorical variables in order to establish correlations between sleep problems and factors such as age, gender, medication use, and lifestyle. T-tests were employed to examine group differences and potential associations among continuous variables, including PSQI ratings, sleep duration, and markers of glycemic control. A significance level of P < 0.05 was deemed to be statistically significant, enabling the detection of important linkages and associations within the gathered data.

Ethical consideration

Ethical considerations were considered, ensuring informed consent from participants and protecting their privacy and confidentiality. The study was conducted in accordance with the Asser Institutional Review Board (H-06-B-091) approved on May 29, 2023 with IRB Log No: REC-10-4-2023.

## Results

Demographic distribution and assessment of sleep disturbances in individuals living with T2D

Table 1 presents the demographic distribution and assessment of sleep disorders on the quality of life and overall well-being of individuals living with T2D. Out of the 230 participants, 52.2% were male (N=110), while 47.8% were female (N=120). In terms of age, the participants were distributed across various age groups, with the largest proportion being in the 45-54 age group (26.1%, N=60), followed by the 55-64 age group (23.9%, N=55). The distribution of participants across the other age groups was as follows: 25-34 (14.3%, N=33), 35-44 (13%, N=30), 64 and older (10.9%, N=25), 18-24 (10.4%, N=24), and under 18 (1.3%, N=3). The highest proportion had been diagnosed with T2D for 1-5 years (29.1%, N=67), followed by 5-10 years (22.2%, N=51). The distribution of participants across the other duration categories was as follows: less than one year (14.8%, N=34), 10-15 years (12.2%, N=28), 15-20 years (12.2%, N=28), and more than 20 years (9.6%, N=22). Most participants reported experiencing difficulties sleeping at night (63.9%, N=147), while the remaining 36.1% (N=83) did not report any sleep problems. In terms of sleep duration, the highest proportion of participants reported getting four to six hours of sleep each night (50.9%, N=117), followed by six to eight hours (29.1%, N=67). A smaller percentage reported getting less than four hours of sleep (13.5%, N=31), while an even smaller proportion reported getting more than eight hours of sleep (6.5%, N=15). Regarding snoring and breathing interruptions, 43.9% of participants (N=101) reported not snoring loudly or experiencing breathing interruptions during sleep, while 21.3% (N=49) reported experiencing these issues. Another portion of participants (34.8%, N=80) was unsure whether they snore or have breathing interruptions during sleep.

**Table 1:** Demographic distribution and assessment of sleep disorders on quality of life and overall well-being in individuals living with type 2 diabetes N=number of respondents; % =percentage

Variable		N	(%)
Gender	Male	110	52.2
	Female	120	47.8
Age	45 – 54	60	26.1
	55 – 64	55	23.9
	25 - 34	33	14.3
	35 – 44	30	13
	Greater than or Equal to 64	25	10.9
	18-24	24	10.4
	Under 18	3	1.4
How long have you been diagnosed with type 2 diabetes?	Less than one Years	34	14.8
	1 - 5 Years	67	29.1
	5 - 10 Years	51	22.2
	10 - 15 Years	28	12.2
	15 - 20 Years	28	12.2
	More than 20 Years	22	9.6
Do you have any problems sleeping at night?	Yes	147	63.9
	No	83	36.1
How many hours of sleep do you usually get each night?	Less than 4 hour	31	13.5
	4 - 6 Hours	117	50.9
	6 - 8 Hours	67	29.1
	More Than 8 Hours	15	6.5
Do you snore loudly or have you been told that you stop breathing while you sleep?	No	101	43.9
	Yes	49	21.3
	Not Sure	80	34.8

Prevalence of sleep-related issues and physical activity

Table 2 presents the prevalence of sleep disorders among individuals diagnosed with T2D, focusing on the frequency of these disorders. The findings reveal important insights into the sleep experiences of the participants. In terms of difficulty in falling asleep, approximately 24.3% reported occasional difficulty, while 21.7% experienced frequent difficulty. Similarly, a notable portion of the participants (24.3%) reported occasionally waking up during the night and having trouble getting back to sleep, while 15.7% experienced this issue on a weekly basis. Excessive daytime sleepiness was reported by 27.0% occasionally and by 14.8% on a more frequent basis. Restless legs or leg muscle cramps that interrupted sleep were experienced occasionally by 16.5% and frequently by 8.7% of the participants. Regarding physical activity, 21.3% engaged in it occasionally, while 15.7% did so on a weekly basis. These findings shed light on the prevalence of sleep disorders in individuals with T2D and emphasize the need for interventions to improve sleep quality and overall well-being in this population. Understanding these sleep patterns can contribute to developing tailored strategies to address sleep disorders and enhance the management of T2D.

**Table 2:** Prevalence of sleep-related issues and physical activity among patients with type 2 diabetes N=number of respondents; %=percentage

	Occasionally (1 - 2 times a month)	(%)	Often (1 - 2 times a week)	(%)	Often (3 or more times a week)	(%)	Infrequently (Less than once a month)	(%)	Never	(%)
How often do you find it difficult to go to sleep?	56	24.3	50.0	21.7	49.0	21.3	48.0	20.9	27.0	11.7
How often do you wake up during the night and have trouble getting back to sleep?	56	24.3	36.0	15.7	41.0	17.8	57.0	24.8	40.0	17.4
How often do you feel excessively sleepy during the day (being very sleepy or sleepy during the day)?	62	27.0	47.0	20.4	34.0	14.8	44.0	19.1	43	18.7
How often do you suffer from restless legs or leg muscle cramps that interrupt your sleep?	38	16.5	35.0	15.2	20.0	8.7	48.0	20.9	89	39.7
How often do you do physical activity for at least 30 minutes, such as [walking - running - cycling - swimming]?	49	21.3	36.0	15.7	35.0	15.2	54.0	23.5	56	24.3

The quality of sleep and various control measures

Table 3 assesses the relationship between the quality of sleep and various control measures in individuals with T2D. The sleep quality is rated on a scale ranging from “Excellent” to “Weak,” with corresponding percentages indicated in each category. The relationship is examined in relation to the use of diabetes medications, types of medication used, regular medication use, and the presence of sleep problems. Regarding the use of diabetes medications, there is no significant association between medication use and sleep quality (p-value = 0.61). Both those using medications and those not using medications reported varying levels of sleep quality across the categories. Similarly, when considering the types of medication used (insulin, cereal, and pills), there is no significant association with sleep quality (p-value = 0.581). The regular use of medications also does not show a significant association with sleep quality (p-value = 0.718). Both those using medications regularly and those not using them reported diverse sleep quality ratings. However, when examining the presence of sleep problems, there is a significant association with sleep quality (p-value = 5.889). Those who reported having sleep problems at night exhibited lower ratings of sleep quality compared to those without sleep problems.

**Table 3:** Assessment of the relationship between quality of sleep and the various control measure in individual with type 2 diabetes Significance at P<0.05

	How do you rate your sleep quality?					
	Excellent	Acceptable	Very Good	Good	Weak	P Value
Are you currently using any medications for diabetes?						
Yes	19 (8.9%)	44 (19.1%)	58 (25.2%)	81 (35.2%)	8 (3.5%)	0.61
No	3 (1.3%)	4 (1.7%)	5 (2.2%)	6 (2.6%)	2 (0.9%)	
Types of medication used						
Insulin	4 (1.7%)	6 (2.6%)	10 (4.3%)	22 (9.6%)	3 (1.3%)	0.581
Cerreal	15 (6.5%)	29 (12.6%)	40 (17.4%)	51 (22.2%)	5 (2.2%)	
Insulin and Pill	3 (1.3%)	13 (5.7%)	13 (5.7%)	14 (6.1%)	2 (0.9%)	
Do you use medications regularly?						
Yes	19 (8.9%)	40 (17.4%)	55 (23.9%)	73 (31.7%)	7 (3.0%)	0.718
No	3 (1.3%)	8 (3.5%)	8 (3.5%)	14 (6.1%)	3 (1.3%)	
Do you have any problems sleeping at night?						
Yes	3 (1.3%)	44 (19.1%)	23 (10.0%)	67 (29.1%)	10 (4.3%)	5.889
No	19 (8.9%)	4 (1.7%)	40 (17.4%)	20 (8.7%)	0	

Overall, the findings suggest that the use of diabetes medications, types of medication, and regular medication use do not have a significant impact on sleep quality in individuals with T2D. However, the presence of sleep problems is associated with lower sleep quality ratings. This highlights the importance of addressing sleep problems to improve overall sleep quality in individuals with T2D.

Sleep-related habits and lifestyle factors among individuals with different durations of T2D diagnosis

Table 4 shows the associations between sleep-related habits, lifestyle factors among individuals with different durations of T2D diagnosis. The risk factors explored include alcohol consumption before bedtime, electronic device usage before bedtime, caffeine consumption four hours before bedtime, and smoking or tobacco product use. Regarding alcohol consumption before bedtime, no individuals diagnosed with T2D for less than one year reported drinking alcohol within two hours before bedtime. However, as the duration of T2D increased, a small percentage of individuals reported consuming alcohol before bedtime. The statistical analysis did not find a significant association between alcohol consumption and the duration of T2D (p-value = 0.466). In terms of electronic device usage before bedtime, a higher proportion of individuals with longer durations of T2D reported using electronic devices an hour before bedtime. The statistical analysis identified a significant association between electronic device usage and the duration of T2D (p-value = 0.002). Regarding caffeine consumption four hours before bedtime, no substantial differences were observed among individuals with varying durations of T2D. The statistical analysis did not indicate a significant association between caffeine consumption and the duration of T2D (p-value = 0.126).

Finally, for smoking or tobacco product use, there were no consistent patterns observed in relation to the duration of T2D. The statistical analysis did not reveal a significant association between smoking or tobacco product use and the duration of T2D (p-value = 0.185). Overall, the findings suggest that electronic device usage before bedtime is associated with the duration of T2D, with individuals diagnosed with longer durations being more likely to use electronic devices before sleep. However, no significant associations were found between the duration of T2D and alcohol consumption, caffeine consumption, or smoking/tobacco product use. These results provide insights into potential risk factors related to the duration of T2D and can guide future studies and interventions aimed at understanding and addressing these factors in individuals with T2D.

**Table 4:** Associations between sleep-related habits, lifestyle factors among individuals with different durations of type 2 diabetes diagnosis Pearson X2 Test @ P<0.05; N=number of respondents; %=percentage: significance level (P<0.05)

	Duration been diagnosed with T2D						
	Less than 1 Year	1 - 5 Years	5 - 10 Years	10 - 15 Years	15 - 20 Years	More than 20 Years	P Value
Do you drink alcohol two hours before bedtime?							
Yes	0	1 (0.4%)	0	0	0	0	0.466
No	32 (13.9%)	65 (28.3%)	51 (22.2%)	28 (12.2%)	28 (12.2%)	22 (9.6%)	
Sometimes	2 (0.9%)	1 (0.4%)	0	0	0	0	
Do you use electronic devices (phone, computer, tablet, etc.) an hour before bedtime?							
Yes	29 (12.6%)	44 (19.1%)	40 (17.4%)	19 (8.3%)	16 (7.0%)	10 (4.3%)	0.002
No	1 (0.4%)	9 (3.9%)	5 (2.2%)	5 (2.2%)	9 (3.9%)	10 (4.3%)	
Sometimes	4 (1.7%)	14 (6.1%)	6 (2.6%)	4 (1.7%)	3 (1.3%)	2 (0.9%)	
Do you consume caffeine (coffee, tea, etc.) 4 hours before bedtime?							
Yes	18 (7.8%)	36 (15.7%)	26 (11.3%)	7 (3.0%)	13 (5.7%)	12 (5.2%)	0.126
No	5 (2.2%)	15 (6.5%)	12 (5.2%)	13 (5.7%)	4 (1.7%)	6 (2.6%)	
Sometimes	11 (4.8%)	16 (7.0%)	13 (5.7%)	8 (3.5%)	11 (4.8%)	4 (1.7%)	
Do you smoke or use tobacco products?							
Yes	11 (4.8%)	18 (7.8%)	11 (4.8%)	6 (2.6%)	2 (0.9%)	3 (1.3%)	0.185
No	23 (10.0%)	49 (21.3%)	40 (17.4%)	22 (9.6%)	26 (11.3%)	19 (8.3%)	

## Discussion

The study revealed a significant prevalence of sleep disturbances among individuals with T2D. Difficulties in falling asleep and waking up during the night were reported by a substantial portion of participants, indicating the presence of insomnia symptoms. Sleep disturbances can significantly impact the overall sleep quality of persons diagnosed with T2D, resulting in fragmented and low-quality sleep. This assertion is further substantiated by the findings of Surani et al. [12]. A significant proportion of participants reported experiencing excessive daytime sleepiness as a prominent symptom. The present discovery indicates that persons diagnosed with T2D may encounter challenges in staying awake and may experience excessive daytime sleepiness. According to Waldman et al. [13], the presence of excessive daytime drowsiness might have detrimental effects on individuals' everyday functioning, such as their productivity and overall quality of life. A lesser proportion of participants reported experiencing the presence of restless legs or leg muscle spasms that disrupt their sleep.

The findings from this study suggest that various factors related to diabetes medications, including the type of medication and regularity of use, do not seem to have a substantial influence on sleep quality among individuals with T2D (Table 3). However, the presence of sleep problems was identified as a significant factor negatively affecting sleep quality. This observation underscores the critical importance of recognizing and addressing sleep issues in individuals living with T2D. The implication here is that managing diabetes medications alone may not be sufficient to improve sleep quality in this population. Instead, healthcare providers should also focus on identifying and treating sleep problems, which can encompass a range of issues such as insomnia, sleep apnea, or restless leg syndrome. According to Lopes et al. [14], poor sleep quality was present in 45% of cases and was associated with age. Addressing these sleep-related concerns may contribute significantly to enhancing overall sleep quality and, in turn, the well-being of individuals with T2D. This finding emphasizes the need for a comprehensive approach to diabetes management that includes the evaluation and management of sleep disturbances as an integral component.

The results also indicate that the study explored the connection between sleep-related habits and lifestyle factors among individuals with varying durations of T2D diagnosis. It's noteworthy that some common lifestyle factors like alcohol consumption, caffeine use, and tobacco product use did not exhibit significant associations with the duration of the disease, as evidenced by p-values greater than 0.05. However, a statistically significant association was found between the use of electronic devices before bedtime and the duration of T2D (p=0.002). This finding implies that while certain lifestyle habits did not appear to be influenced by the duration of T2D, the use of electronic devices before bedtime showed a clear correlation. It could suggest that prolonged exposure to electronic screens before sleep may have specific implications for individuals with T2D, potentially affecting their sleep patterns or overall health. These results suggest that other factors not included in the study may contribute more significantly to sleep disturbances in individuals with T2D. The result was however contrary to what was reported by Yeh [15] that 1,254 smoking adults developed T2D compared with adults who never smoked.

A substantial portion of participants reported difficulties in falling asleep and waking up during the night. These symptoms are indicative of insomnia, a common sleep disorder, which is known to be more prevalent in individuals with chronic health conditions like T2D [16]. The presence of insomnia symptoms can lead to fragmented and poor-quality sleep. This fragmented sleep pattern can result in individuals not getting enough restorative sleep, which can have negative effects on their overall health and well-being [17]. Many participants reported experiencing excessive daytime sleepiness. This could be due to the poor quality of sleep at night, leading to difficulties in maintaining wakefulness during the day. Daytime sleepiness can affect daily functioning, productivity, and quality of life.

Restless legs or leg muscle cramps that interrupt sleep were experienced by a notable percentage of participants. These symptoms can disrupt sleep continuity and contribute to sleep fragmentation. Restless legs syndrome is known to be more prevalent in individuals with certain medical conditions, including diabetes [18]. The study did not find a significant association between the duration of T2D and sleep disturbances. This suggests that sleep problems may be prevalent across different stages of the disease. The chronic nature of diabetes itself, along with associated factors like blood sugar fluctuations, could contribute to sleep disturbances. While the study did not identify significant associations with factors like alcohol consumption, caffeine consumption, or smoking, it's possible that other unexamined factors could be contributing to sleep disturbances. Diabetes-related factors such as medication side effects, neuropathy (nerve damage), and metabolic changes could play a role. The study found a significant association between the presence of sleep problems and lower sleep quality ratings, emphasizing the impact of sleep disturbances on overall well-being and quality of life.

The high prevalence of sleep disturbances in individuals with T2D can be attributed to various pathophysiological mechanisms and interactions though the exact cause of sleep issues in diabetes is complex and multifactorial. Insulin resistance, a hallmark of T2D, can disrupt the regulation of glucose in the body. Fluctuations in blood glucose levels, particularly during the night, can disturb sleep. High glucose levels can lead to increased urination (nocturia), causing individuals to wake up frequently during the night [19]. Restless legs syndrome, which is prevalent among the respondents is a symptom of diabetic neuropathy, a common complication of T2D, which can lead to nerve damage and sensory abnormalities [20]. This neuropathy can cause uncomfortable sensations, such as tingling, burning, or pain in the legs, which may be worse at night [21]. These sensations can result in restlessness and difficulty staying asleep, contributing to sleep disturbances. OSA and obesity is a major risk factor for both T2D and is characterized by repeated episodes of disrupted breathing during sleep. The excess weight and fat deposits around the neck can lead to airway obstruction, causing brief awakenings throughout the night [22].

The combination of T2D and OSA can exacerbate sleep problems and lead to daytime sleepiness. T2D can lead to hormonal imbalances, including disruptions in the secretion of insulin and other hormones. These imbalances can affect the body's internal clock (circadian rhythm) that regulates sleep-wake patterns [23]. Managing a chronic condition like T2D can lead to increased stress, anxiety, and depression. Psychological factors can significantly impact sleep quality by causing racing thoughts, difficulty relaxing, and heightened arousal, all of which interfere with falling asleep and staying asleep [24]. Diabetes can result in heightened sympathetic nervous system activity, which is responsible for the “fight or flight” response. Increased sympathetic activity at night can lead to elevated heart rate and blood pressure, making it challenging to relax and fall asleep [25].

This study has some limitations, including the use of self-reported measures to assess sleep disorders, which could lead to recall bias and subjectivity in participant replies. Data on sleep patterns may be more trustworthy and accurate when derived from objective tests like polysomnography or actigraphy. A cross-sectional design captures data at a single point in time, which makes it challenging to establish causal relationships. It is difficult to determine whether sleep disturbances cause certain outcomes or whether other factors contribute to both sleep disturbances and the outcomes being studied. Since participants were recruited through online platforms, the findings might not be generalizable to individuals who do not use the internet or participate in online diabetes communities. The demographic and health characteristics of the online community might differ from the broader population of individuals with T2D.

## Conclusions

This research underscores the significant impact of sleep disturbances on the well-being of individuals with T2D, emphasizing the urgent need for healthcare providers to proactively address these issues as part of comprehensive diabetes care. While the study reveals that the effect of alcohol consumption on sleep disturbances remains consistent regardless of disease duration, it suggests unexplored factors may contribute more substantially. Although no strong link is established between T2D duration and smoking habits, the study highlights the negative influence of sleep disruptions on overall health. Implementing tailored interventions, including personalized sleep hygiene recommendations, stress management strategies, and potential collaboration with sleep specialists, could offer effective avenues to improve sleep quality and overall health outcomes for those with T2D. Further research is recommended to uncover additional contributing factors.
